# Angioleiomyomas in the head and neck: A retrospective clinical and immunohistochemical analysis

**DOI:** 10.3892/ol.2014.2124

**Published:** 2014-05-08

**Authors:** YING LIU, BO LI, LONGJIANG LI, YANBIN LIU, CHENXING WANG, LAGABAIYILA ZHA

**Affiliations:** 1 Department of Oral and Maxillofacial Surgery, Xiangya Stomatological Hospital, Central South University, Changsha, Hunan, P.R. China; 2 State Key Laboratory of Oral Diseases, West China Hospital of Stomatology, Sichuan University, Chengdu, Sichuan, P.R. China; 3 Department of Head and Neck Oncology Surgery, West China Hospital of Stomatology, Sichuan University, Chengdu, Sichuan, P.R. China; 4 Department of Forensic Science, School of Basic Medical Sciences, Central South University, Changsha, Hunan, P.R. China

**Keywords:** angioleiomyomas, head and neck region, retrospective study, nerve, immunohistochemical analysis

## Abstract

Angioleiomyoma is a benign soft-tissue tumor originating from vascular smooth muscle, and is rare in the head and neck. The present study retrospectively examined a cohort of patients with head and neck angioleiomyoma treated at the West China Hospital of Stomatology, and also subjected archived tissues to modern immunohistochemical analysis. In total, 21 patients were treated for angioleiomyoma between 1978 and 2012 at the West China Hospital of Stomatology, Sichuan University (Chengdu, Sichuan, China). Medical records were examined and paraffin block sections were cut and stained with hematoxylin and eosin, Masson’s trichrome stain and Van Gieson stain, prior to being subjected to immunohistochemical analysis to re-evaluate and confirm the diagnoses. Angioleiomyomas were found to account for only 0.18% of the benign head and neck tumors in the patients presenting to the hospital over the past 34 years. The diagnosis was more common in males (male:female ratio, 1.625:1) and the mean age at diagnosis was 42.5 years. The most common sites were the buccal mucosa, parotid gland and palate. More than half of the tumors (61.9%) were >2 cm in diameter. Five tumors presented with pain and/or tenderness. The histological subtype was reported as solid in five cases, venous in six, cavernous in nine and venous-cavernous in one. Three tumors exhibited nerve neurofibrils. All tumors were excised with no subsequent recurrence. Cytological and imaging examinations were not useful for pre-operative diagnosis. Angioleiomyoma is a benign tumor that causes limited morbidity. Surgical excision is the only effective treatment and recurrence is rare. The present study revealed that nerves were present in a small proportion (14.3%) of tumors. It was hypothesized that the compression of nerves accompanying numerous blood vessels in the tumor may cause pain, particularly in venous- and cavernous-type angioleiomyomas.

## Introduction

Angioleiomyoma, also termed vascular or dermal angiomyoma, is an uncommon benign soft tissue tumor (1–3), comprising approximately two-thirds of oral leiomyomas reported in previous studies (4,5). The rarity of the tumor is likely due to the paucity of smooth muscle in the oral cavity, the primary source of smooth muscle being the tunica media of blood vessels (6). The tumor normally manifests as a painful solid mass on the extremities (in 89.0% of cases), particularly below the knee; only 8.5% arise in the head and neck (7,8). At present, <200 head and neck angioleiomyomas have been previously reported, and they have tended to present as painless masses of the venous or cavernous type (1,7). Angioleiomyoma is difficult to diagnose correctly from clinical manifestations and imaging alone, and a biopsy is generally required (1,9).

Angioleiomyomas comprise vascular endothelial and smooth muscle cells (2,10). Morimoto classified the tumors as follows (7): i) Solid, the tumor comprises closely compacted smooth muscle and abundant blood vessels, which are small and slit-like. The smooth muscle bundles surround the vessels and interdigitate with them; ii) venous, the tumor lacks compacted smooth muscle bundles and the blood vessels have thick muscular walls of varying size; iii) cavernous, the tumor consists of numerous dilated vascular channels and smaller quantities of smooth muscle bundles, which are difficult to distinguish from the muscular walls of the vessel channels.

The present study retrospectively examined the clinical features, characteristics and management of all head and neck angioleiomyomas that were treated between 1978 and 2012 at the West China Hospital of Stomatology, Sichuan University (Chengdu, Sichuan, China). Where possible, archived tissue was subjected to modern immunohistochemical diagnostic techniques.

## Patients and methods

Patient records and analysis. In total, 21 patients have been diagnosed with angioleiomyoma at the West China Hospital of Stomatology over the past 34 years. The records of these patients were examined and recorded, including data on age, gender, presenting symptoms and their duration, clinical features, investigations and method of diagnosis, tumor location and size, original diagnosis and Morimoto’s pathological classification, and subsequent management and surveillance (7). Archived paraffin blocks were cut and stained with hematoxylin and eosin, Masson’s trichrome stain, Van Gieson stain and immunohistochemical stain (avidin-biotin complex method) to re-evaluate and confirm the diagnosis. For the immunohistochemical analysis, anti-vimentin goat polyclonal (sc-7557; Santa Cruz Biotechnology Inc., Santa Cruz, CA, USA), anti-CD34 mouse monoclonal (sc-7324, Santa Cruz, Biotechnology, Inc.), anti-S-100 rabbit polyclonal (ab868; Abcam, Cambridge, UK), anti-desmin rabbit polyclonal (ab32362; Abcam) and anti-α-actin smooth muscle rabbit polyclonal (ab5694; Abcam) antibodies were used. The present study followed the Declaration of Helsinki for the medical protocol and was approved by the regional Ethical Review Board of the West China Hospital of Stomatology.

## Results

### Distribution

The patients diagnosed with angioleiomyomas accounted for 0.18% (21 out of 11,916) of all benign head and neck tumors, 0.17% (four out of 2,413) of all benign salivary gland tumors and 91.3% (21 out of 23) of all leiomyomas during the 34-year study period.

### Clinical features

The clinical characteristics of the present cohort and details of the disease are shown in Table I. Those diagnosed with angioleiomyoma were predominantly male (male:female ratio, 1.625:1), with a mean age of 42.5 years. Approximately half (52.4%) of cases were diagnosed in the fourth to sixth decades. Six tumors were detected in the deep tissues, including four in the parotid gland, one in the mandible and one in the neck. The remaining tumors were located in the submucosal layer (12 cases) or the dermis/subcutaneous layer of the skin (three cases). Tumors tended to be solitary, mobile, firm, ovoid and well circumscribed, with a smooth surface; only two were soft. Macroscopically, certain tumors were brown with hemorrhagic areas on the surface. Others were light red, similar to muscle. One patient had a painless ulcer on the tumor surface (0.3 cm diameter). The majority of patients reported no pain, with the exception of three patients who exhibited spontaneous pain and three with tenderness; one reported pain and tenderness (Table II). Two of the patients who reported pain were also found to have nerve tissue within the tumor (cases 8 and 20).

### Investigations

**Figure 1:**
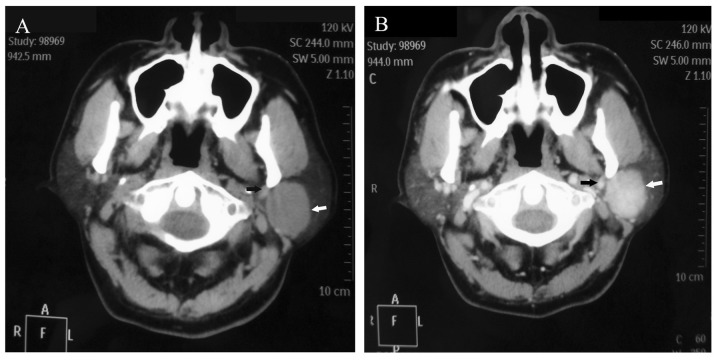
Imaging examination of the angioleiomyoma in the left parotid gland. (A) Computed tomography scan showing an ovoid lesion with a regular margin in the left parotid gland (white arrow), displacing the posterior facial vein (black arrow). (B) Enhanced computed tomography scan showing an ovoid, high-density lesion with heterogeneous enhancement (white arrow), together with enhancement of the posterior facial vein (black arrow).

Fine-needle aspiration (FNA) was undertaken in five patients, and revealed blood and cast-off cells, indicating the presence of hemangiomas. Enhanced computed tomography (ECT) of the head and neck was performed in three cases and Doppler ultrasonography in seven. An example of an ECT image is shown in Fig. 1, showing an ovoid, high-density area with a regular margin, but heterogeneous enhancement displacing the left posterior facial vein in the parotid gland. In this case, color Doppler examination also revealed high resistance to blood flow in the intratumoral arteries, indicating that the tumor was benign. In one case, Doppler ultrasonography detected a feeding artery supplying an angioleiomyoma in the parotid gland from the superficial temporal artery. In four cases, color Doppler flow imaging did not detect continuous or intermittent blood flow. Digital radiography of a tumor in the mandible revealed a cystic lesion with clearly defined borders in the left angle extending from the distal root of D6 to the middle of the mandibular ramus, subsequently leading to tooth mobility of D7 and D8. None of the cases were diagnosed correctly by FNA or imaging studies.

### Treatment and follow-up

All patients underwent excision of the tumor. Those with tumors in the parotid gland also underwent parotidectomy. Intraoperatively, the tumors were found to be well circumscribed. One tumor had a partial capsule, however, the remainder had complete capsules. Their cut surfaces were gray-white, brown or dark red. The follow-up duration was 6–81 months, during which, no local recurrences were observed (although two patients were lost to follow up).

### Pathological findings

**Figure 2:**
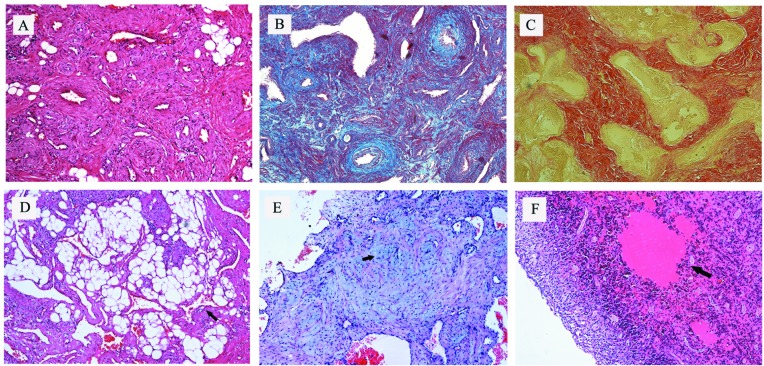
Angioleiomyoma tissue stained with H&E, Masson’s trichrome and Van Gieson stains. (A) The angioleiomyoma comprised of rich vascular channels with thick vessel walls and smooth muscle bundles with elongated nuclei (magnification, ×100). (B) Masson’s stain showing smooth muscle fibers in red, erythrocytes in blue, collagen fibers in black and nuclei in black-blue (magnification, ×100). (C) Van Gieson stain showing smooth muscle fibers in red and collagen fibers in yellow (magnification, ×100). (D) Groups of mature fat cells observed by H&E staining (magnification, ×100). (E) Focal myxoid change (black arrow) in the lesion, stained blue with H&E staining (magnification, ×100). (F) Hyaline change (black arrow) observed by H&E staining (magnification, ×100). H&E, hematoxylin and eosin.

Microscopically, the lesions consisted of a proliferation of smooth muscle cells and numerous blood vessels of varying size that were surrounded by smooth muscle bundles. The spindle-shaped cells were characterized by elongated, cigar-like nuclei and an eosinophilic cytoplasm. Smooth muscle bundles with collagen fibers exhibited an interlacing and swirling arrangement. The abundant blood vessels, with thin or thick walls and circular, slit-like or stellate lumens, were of varying size between tumors and in different areas of the same tumor. In certain cases, neoplastic cells were arranged around vessels that were lined with endothelial cells containing basophilic nuclei. Out of the 21 tumors, five were solid, six venous, nine cavernous and one venous-cavernous (Table III). It was noteworthy that six of the tumors (four solid and one each of the venous and cavernous types) contained focal areas with hyaline change. In eight tumors, including venous and cavernous lesions, focal myxoid change was present. Areas containing small groups of mature fat cells were observed in three tumors, one each of the venous, cavernous and venous-cavernous types (Fig. 2). Two solid tumors demonstrated calcified foci. Focal lymphocytic infiltration was identified in 11 tumors. Neither thrombi nor hemosiderin deposits were observed in any of the tumors.

**Figure 3:**
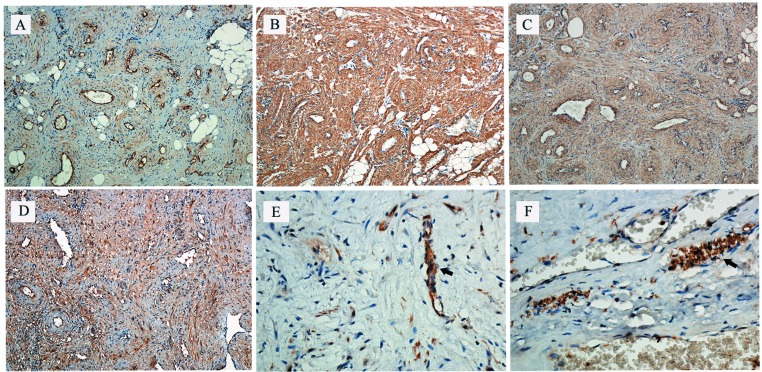
Immunohistochemical characteristics of head and neck angioleiomyoma. (A) Positive staining with anti-CD34 antibody exhibited by endothelial cells that lined the vascular spaces within the tumor (magnification, ×100). (B) Diffuse positive staining with anti-α-actin smooth muscle antibody showing spindle-shaped cells in smooth muscle cell bundles in the tumor (magnification, ×100). (C) Positive staining with anti-vimentin antibody showing expression in the cytoplasm of spindle-shaped cells (magnification, ×100). (D) Positive staining with anti-desmin antibody showing expression in the cytoplasm of neoplastic cells in ALM (magnification, ×100). (E) Positive staining with anti-S100 antibody showing nerve tissue near the capsule (black arrow; magnification, ×400). (F) Positive staining with anti-S100 antibody showing occasional tiny neurofibrils, closely associated with vessels (black arrow; magnification, ×400).

Immunohistochemical analysis revealed that the spindle-shaped tumor cells were diffusely reactive to α-actin smooth muscle and vimentin, and focally reactive to desmin. Endothelial cells in tumor vessels stained positively for CD34. In three cases, a few small neurons that stained positively for S-100 were evident close to the capsule (two cases) or interstitium (one case; Fig. 3). The diagnosis of angioleiomyoma was confirmed on the basis of these findings.

## Discussion

Angioleiomyomas are extremely rare tumors, particularly in the head and neck. The 21 patients included in the present study who were treated over a period of 34 years account for 10% of the cases in the published literature. The present cohort was significantly younger than those investigated in other studies, with a mean age of 42.5 years at diagnosis and a peak in presentation in the fourth to sixth decades. Angioleiomyomas are particularly rare in children (1,3,11–14) and there is only one study of a congenital tumor (15). In the present study, the youngest patient was 10 years old, with an angioleiomyoma located in the mandible. Previous studies have reported that the lip, palate and tongue are the most common sites for angioleiomyomas in the oral cavity (3,14,16), however, the present study identified a preponderance of tumors in the buccal mucosa, followed by the parotid gland and palate. The majority of angioleiomyomas are <2 cm in diameter on presentation and rarely exceed 4–5 cm (1,3,8,13), however, more than half the tumors in the present cohort measured >2 cm in diameter at diagnosis. These differences may be explained by referral patterns to the specialist West China Hospital of Stomatology and the population it serves. In addition, the four parotid angioleiomyomas are noteworthy, as these tumors are particularly unusual in the major salivary glands. Currently, <15 cases (including the four in the present study) have been previously reported (5,13,17–21).

In addition to the three classical pathological types, Morimoto further classified angioleiomyomas into two groups: i) A larger group of extremity tumors that are frequently painful and predominantly of the solid type; and ii) a smaller group of painless tumors on the head that are predominantly (75%) of the venous type (7). If pain is provoked by angioleiomyomas, it is occasionally described as paroxysmal, stinging or radiating, which are essentially the symptoms of neuropathic pain (7,22,23). One previous study indicated that only a small proportion (7.8%) of oral angioleiomyomas are painful (22). In the present study, three patients had radiating or stinging pain reminiscent of neuropathic pain, three reported tenderness and one reported pain provoked by exposure to the cold. Other studies have described pain that can be provoked by cold and wind, pressure, pregnancy and menstruation, however, it can also be spontaneous (1,3,9,24). There are three possible theories explaining the cause of the pain: i) The contraction of smooth muscle vessels, particularly those in solid-type lesions, may cause local ischemia (7); ii) the compression of nerves accompanying the blood vessels in the tumor (24), which relies on the existence of neurofibrils within the tumor capsule (1,12,14,25,26); and iii) secondary inflammation of the mass, with a mild to moderate infiltration of inflammatory cells (22). At present, the first two theories are considered to be the most plausible. Fox *et al* found small neurofibrils, some closely associated with vessels, between the smooth muscle bundles within the body of the tumor, but argued that the capsular neurofibrils were more likely to be pain generators than the small number of interstitial neurofibrils (27). The present study revealed positive immunoexpression of S-100 antigen by neurofibrils in three tumors (of venous and cavernous type), indicating that the presence of small interstitial neurofibrils and capsular nerves may explain the occurrence and nature of the pain in certain patients (3). In the present study, extensive focal lymphocytic infiltrations were also found in two tumors, although in one of these cases the patient did not report pain. It was hypothesized that compression of the nerves accompanying numerous blood vessels in the tumor causes the pain, particularly in venous-type and cavernous-type angioleiomyomas. In other words, the pain may be closely associated with nerve and pathological type. Although it was hypothesized that the presence of neural tissue in an angioleiomyoma may be responsible for the symptoms of neuropathic pain, the sample size used in the present study is too small to draw any firm conclusions. Further studies are required, although accumulating a sufficiently large cohort on which to perform meaningful statistical analysis is a challenge in such a rare tumor.

The pre-operative diagnosis of head and neck angioleiomyomas is challenging (1). FNA and cytology were not beneficial in the present study, indeed it provided a misdiagnosis of hemangioma or schwannoma on several occasions. Although imaging was also unhelpful in this regard, it at least yielded information that guided the surgery, particularly in those patients who also required parotidectomy. Magnetic resonance imaging (MRI) can distinguish fibrous nodules (low signal intensity on T1-and T2-weighted images) and lipomas (high signal intensity on T1-weighted images) from angioleiomyomas (28), which show a uniform signal pattern, with T1 signal intensity partially greater than the surrounding soft tissue, but with marked hyperintensity on T2-weighted sequences (29). Nevertheless, none of the patients in the present study underwent MRI.

The differential diagnosis of angioleiomyoma includes vascular tumors (such as hemangiomas and lymphangiomas), certain benign mesenchymal tumors (including lipomas, schwannomas and neurofibromas), pleomorphic adenomas and cysts (1,3,9). Histological examination following resection remains the most reliable method for diagnosis. In the present study, Masson’s trichrome stain, Van Gieson stain and the positive expression of desmin and α-actin smooth muscle antibodies demonstrated the presence of smooth muscle cells, and the positive expression of CD34 demonstrated the presence of vascular endothelium. It is important to differentiate angioleiomyoma from other types of spindle cell tumor, including leiomyoma (CD34^−^ and S-100^−^), myofibroma (desmin^−^, CD34^−^ and S-100^−/+^) and myopericytoma (desmin^−^, CD34^−^ and S100^−^) (11). It is particularly important to distinguish angioleiomyomas from malignant mesenchymal tumors, including leiomyosarcoma (30). Angioleiomyomas are predominantly composed of mature smooth muscle cells, while leiomyosarcomas consist mainly of undifferentiated mesenchymal cells or fibroblast-like cells and myofibroblast-like cells (31). Immunohistochemical and molecular markers, for example, proliferating cell nuclear antigen, B-cell lymphoma 2, cyclin-dependent kinase 4, p53 and mouse double minute 2 homolog also allow accurate discrimination between benign and malignant smooth muscle tumors (32). A strength of the present study was the access to archived tissue, which allowed tissue collected up to 34 years ago to be subjected to modern immunohistochemical analysis, thus adding substantially to the body of literature on these rare tumors.

In the present study, one patient had undergone laser therapy at a local hospital prior to referral, however, the tumor recurred one year later. In accordance with the literature, the present study concluded that surgical resection along the tumor capsule is the most effective treatment for all head and neck angioleiomyomas (1,9,13), as recurrence is rare following resection (13,33). To the best of our knowledge, there have been no recurrences among the patients enrolled in the present study, although two were lost to follow-up. Other studies have reported four head and neck angioleiomyomas that have recurred following resection: One in the external skin of the nose at eight years; one in the larynx at an unspecified time; and two recurring one and seven years after resection, although the location was not reported (13,34,35).

In conclusion, it is extremely rare for an angioleiomyoma to present in the head or neck; such cases comprised only 0.18% of the cases at the West China Hospital of Stomatology, a specialist institution, over 34 years. The diagnosis could only be made pre-operatively if a biopsy was undertaken; pre-operative cytological and imaging investigations were not fruitful in any of the cases. At present, surgical excision is the only effective treatment and recurrence is rare. In the present study, three tumors that contained neurofibrils were identified. Angioleiomyoma is a benign tumor and surgery is almost always curative, and although it is rare in the head and neck, it is important to recognize that it can only be reliably diagnosed prior to surgery by biopsy.

## Figures and Tables

**Table I:** Characteristics of the 21-patient cohort.

Characteristic	Value (%)
Gender	
Male	13 (61.9)
Female	8 (38.1)
M/F ratio	1.625:1
Age, years	
Range	10–65
Mean	42.5
Males	42.4
Females	42.6
Site	
Buccal mucosa	6 (28.5)
Parotid gland	4 (19.0)
Palate	4 (19.0)
Nasolabial groove	2 (9.5)
Lip	1 (4.8)
Tongue	1 (4.8)
Gingiva	1 (4.8)
Mandible	1 (4.8)
Neck	1 (4.8)
Diameter, cm	
≤2	8 (38.1)
>2	13 (61.9)
Symptoms	
Painless	16 (76.2)
Tender	2 (9.5)
Spontaneously painful^a^	3 (14.3)
Duration	
Longest, years	30+
Shortest, months	1+

a One patient exhibited pain and tenderness.

**Table II:** Summary of clinical information of the patient cohort.

Case	Age, years	Gender	Tumor location	Size, cm	Duration	Symptoms	Examination	Imaging	Pre-operative diagnosis	Treatment	Recurrence
1	62	F	Buccal mucosa	1.5	15 years	Painless	None	CDU	Hemangioma	Excision	NR
2	46	M	Parotid gland	3.2	1 year	Painless	None	ECT/CDU	Pleomorphic adenoma	LSD	NR
3	38	F	Parotid gland	2.5	8 months	Painless	None	ECT	Pleomorphic adenoma	LAD	NR
4	49	M	Buccal mucosa	2.0	1 year	Painless	None	CDU	Unknown mass	Excision	NR
5	36	F	Buccal mucosa	2.0	1 year	Painless	None	ECT	Unknown mass	Excision	NR
6	65	F	Nasolabial groove	2.5	30 years	Painless	FNA-blood	CDU	Schwannoma	Excision	NR
7	51	M	Parotid gland	3.5	3 months	Painless	None	ECT/CDU	Pleomorphic adenoma	LAD	NR
8	51	F	Buccal mucosa	1.0	6 years	Pain	None	None	Unknown mass	Excision	NR
9	50	M	Nasolabial groove	3.0	18 years	Painless	FNA-blood	ECT	Hemangioma	Excision	NR
10	49	F	Palate	1.0	3 months	Painless	FNA-blood, cast-off cells	None	Hemangioma	Excision	NR
11	42	M	Parotid gland	2.4	1 year	Painless	None	CDU	Pleomorphic adenoma	Excision	NR
12	10	F	Mandible	3.5	1 month	Painless	Biopsy	DR	Unknown cyst	Excision	NR
13	30	F	Buccal mucosa	3.0	1 month	Painless	FNA-blood	CDU	Unknown mass	Excision	NR
14	20	M	Gingiva	2.5	1 month	Tenderness	Biopsy	None	Mass	Excision	NR
15	60	M	Palate	1.5	1 year	Painless	None	None	Pleomorphic adenoma	Excision	NR
16	34	M	palate	1.0	5 years	Painless	None	None	Unknown mass	Excision	NR
17	58	M	Lip	6.0	15 years	Pain	None	None	Neurofibroma	Excision	NK
18	18	M	Palate	3.5	2 month	Painless	FNA-blood	None	Pleomorphic adenoma	Excision	NR
19	19	M	Tongue	3.5	2 years	Tenderness	None	None	Unknown mass	Excision	NR
20	47	M	Buccal mucosa	2.0	2 month	Pain Tenderness	Biopsy	None	Mass	Excision	NK
21	57	M	Neck	4.0	4 years	Painless	FNA-blood	None	Schwannoma	Excision	NR

M, Male; F, Female; FNA, fine-needle aspiration; CDU, color Doppler ultrasonography; ECT, enhanced computed tomography; DR, digital radiography; LSD, local resection + superficial lobe parotidectomy + dissection of facial nerve; LAD, local resection + all lobe parotidectomy + dissection of facial nerve; NR, no recurrence; NK, unknown (lost to follow-up).

**Table III:** Summary of pathological characteristics of the 21 tumors.

	Microscopic					
	
Histological type	Hyaline change	Myxoid change	Mature fat cell	Foci of calcification	Focal lymphocytic infiltration	Nerves
Solid, n	4	0	0	2	4	1
Venous, n	1	2	1	0	6	1
Cavernous, n	1	5	1	0	1	1
Venous-cavernous, n	0	0	1	0	0	0
Total, n (%)	6 (28.6)	7 (38.1)	3 (14.3)	2 (9.5)	11 (52.4)	3 (14.3)
